# Working in a minefield; Nurses’ strategies for handling medicine administration interruptions in hospitals, -a qualtiative interview study

**DOI:** 10.1186/s12913-021-07122-8

**Published:** 2021-10-14

**Authors:** Johanne Alteren, Marit Hermstad, Lisbeth Nerdal, Sue Jordan

**Affiliations:** 1 Molde University College, Faculty of Health Sciences and Social Care, Britvegen 2, 6410 Molde, Norway; 2 Helgeland Hospital Trust, Prestmarkveien 1, 8800 Sandnessjøen, Norway; 3 Nord University Helgeland, Faculty of Nursing and Health Science, Torggata 5, 8622 Mo I Rana, Norway; 4 Department of Nursing, Swansea University, Singleton Park, Sketty, Swansea, Wales SA2 8PP UK

**Keywords:** Hospitals, Medicine management, Registered nurses, Patient safety, Management, Work organisation, Work interruptions, Medication errors

## Abstract

**Background:**

Administering medicines is one of the most high-risk tasks in health care. However, nurses are frequently interrupted during medicine administration, which jeopardises patient safety. Few studies have examined nurses’ experiences and the strategies they adopt to cope with interruptions during medicine rounds. This paper identifies nurses’ strategies for handling and reducing interruptions and ensuring safety during medicine rounds, within the confines of the hospitals’ organisational systems.

**Methods:**

This descriptive and exploratory research study was undertaken with experienced nurses in Norwegian hospitals in 2015 using semi-structured interviews. Interviews were designed to elicit experiences and strategies used for handling interruptions to medicine rounds. Data were analysed using qualitative content analysis based on inductive reasoning to identify meaningful subjects and reach an interpretive level of understanding regarding nurses’ experiences.

**Results:**

All 19 senior nurses who were approached were interviewed. From 644 condensed meaning units, we identified eight interpretative units and three themes: ‘working in environments of interruptions’, ‘personal coping strategies’, and ‘management-related strategies’. Nurses’ working environments were characterised by interruptions and distractions, which often threatened patient safety. To handle this unpredictability and maintain ward organisation, nurses developed their own personal strategies to overcome inherent problems with their working conditions, the absence of effective management, and colleagues’ reluctance to assume responsibility for minimising interruptions.

**Conclusions:**

Administration of medicines in hospitals can be described as ‘working in a minefield’. Our findings indicate that the hospital management, in cooperation with nurses and other healthcare professionals, should take responsibility for improving the routine process of medicine administration by minimising avoidable interruptions. Patient safety can be improved when the hospital management takes steps to protect nurses’ work environments and assumes responsibility for resolving these challenges.

## Background

Nurses worldwide are frequently interrupted when administering medicines in surgical and medical wards [1–4]. Interruptions lead to errors and threaten patient safety [1, 5, 6], and result in patient harm [7]. The threat to patient safety from medication errors has been increasing in parallel to the use of medicines [8]. The World Health Organisation (WHO) [9] aims to reduce severe avoidable medication-related harm by 50% globally by 2022 by targeting health care professionals, patients, the work environment, medicines, tasks, computerised information systems, the primary-secondary care interface, and high-risk situations. Systematic reviews of empirical evidence of the causes of medication errors in hospital settings [10, 11] have identified information exchange, conversations, and alarm systems as the most common underlying causes of unsafe acts [11].

Reviews of the effectiveness of strategies aimed at reducing interruptions during medicine administration indicate that educational strategies, organisational strategies such as creation of quiet zones, use of checklists and vests, and new technologies may minimise interruptions [12, 13]. However, there is either very limited [13] or no [14] evidence on existing strategies being effective in reducing medication errors or patient harm. Work interruptions (WIs) due to direct patient care or system failures, such as missing medicines, are common and frequently cause problems during medicine rounds [1, 7]. WIs generally have negative consequences on patients’ safety and outcomes, employees’ well-being and performance, as well as a country’s resources [7, 9].

Nurses are rarely able to complete nursing activities without being interrupted by questions, complaints, statements, double-checks, and alarms [1, 15, 16], particularly when concentration is most needed to prevent errors [1, 5]. The most common sources of interruptions during medicine administration are nursing colleagues, other staff, and nurses themselves performing other activities [1, 17, 18]; interruptions from patients and telephone calls seemed to be the most problematic [17].

Regardless of the unit, time of day, or day of the week, medicine administration entails a complex mix of varied and often competing or conflicting demands [19]. Unsafe actions and errors are influenced by workload; staff shortage; local working conditions [10] such as noise and lighting [10]; the configuration, features and supervision of the ward-based medicine system; nurses’ management of interruptions and distractions including emergencies and chaotic environments; and nurses’ interaction with patients [20]. However, experienced, senior nurses learn to protect the patient by renewing their focus and continuing to administer medicine [1]. They are capable of dividing their attention, for example, they can simultaneously walk and make patient-related decisions, administer medicine whilst answering the phone, notice another patient’s physicians and decide to engage with them or answer the phone while administering intravenous medicine [21, 22].

Nurses individualise their coping strategies and techniques, either by multi-tasking, engaging with the task [19, 21, 22], or focusing solely on patient interactions [20], depending on the complexity of the task and their nursing experience [23]. Some nurses use the medicine round as an opportunity to interact with their patients in addition to the administration of medicines [20]. Other nurses prioritise managing time, particularly when handling interruptions [19]. In addition to their own strategies, nurses are also required to adhere to the organisations’ expectations of how interruptions need to be handled [22]. Maximising patients’ satisfaction could militate against patient safety. For example, nurses must judge when it is more important to stop to answer a call light, as against administer the medicine on time [22].

Medicine administration is considered inseparable from other nursing work [21] and should be contextualised within the organisation of clinical care and medicine optimisation [24]. Few interruptions are related to medicine related tasks [1, 4], indicating considerable scope to reduce unnecessary interruptions [4]. This study aimed to explore this and examine how nurses handle interruptions during medicine rounds to gain a deeper understanding of their experiences of working in environments where they are frequently interrupted and are required to adopt strategies to ultimately improve safety in medicine administration.

## Methods

### Design

This study employed a descriptive and exploratory design based on qualitative and interpretative analysis of semi-structured interviews.

### The context of the study

The nurses who participated in this study worked in three local hospitals serving approximately 77,000 inhabitants in 18 municipalities [1]. In Norway, the hospitals are organised into three levels: local, central, and regional.

A head nurse working the dayshift managed each ward. They were responsible for management, professions, employees, and finance. The total number of patient beds were 56, with 22 and 17 in each of the surgical wards and 17 in the medical ward. The wards were organised into two teams. In each team, one nurse was responsible for administering medicines to about 10 patients. As per the current practice, nurses worked shifts of 8 h with intense, concentrated work periods, which included a wide range of duties as well as several medicine rounds. The number of health personnel on duty for the day and evening shifts differed (Table 1). Nurses obtain a 3-year university education, and nurse assistants obtain a 1- to 3-year education from high school or trade school.

**Table 1:** Health personnel on duty in each shift

	Day shift		Evening shift	
	Nurses	Nurse assistants	Nurses	Nurse assistants
**Surgical ward 1**	5	3–4	3	2
**Surgical ward 2**	3 or 4	2 or 1	2	2
**Medical ward**	3 or 4	3 or 2	2	2

Normally, medicines are stored in a medicine room where nurses prepare them at designated times (8.00 am, 12.00 noon, 3.00 pm, and 6.00 pm). At this stage, medicines are checked thoroughly; nurses arrange and double-check the medicines for the next day and examine patient allergy statuses from their notes. When double-checking, two nurses verify the medicines in the dose distribution system against the prescription and sign the medicine journal. Medicines are administered to one patient at a time from a drug trolley using a unit-dose distribution system.

### Data collection

The study protocol was approved by an ethics committee at the Norwegian Centre for Research Data (project number 30223). The researchers requested participation, verbally and in writing, from the nurses in charge of the surgical wards in two of the hospitals and the medical ward from the third hospital (which has no surgical ward). The head nurses informed all 58 nurses working in the wards during the day and evening shifts about the study’s purpose and procedures, distributed the written information, discussed the relevant ethical issues, and requested volunteers. They received assurance that participation was voluntary, and they could withdraw from the study whenever they wanted to without consequences and having to explain why. The nurses who participated, signed informed consent forms, after which the study commenced [1]; no one withdrew from participation during the study. The nurses were interviewed to gain a deeper understanding about working in environments where they were frequently interrupted when administering medicines.

After we scheduled the interviews, we grouped the nurses according to their wards and assigned each nurse a number. We selected nurses to be interviewed and the order for the interviews by drawing lots. Of importance to this study was not how much data the researchers could gather, or from how many sources data could be collected, but whether the data collected would be sufficiently rich to refine and clarify our understanding of the nurses’ experiences [25].

Two university researchers and one hospital nurse (co-researcher) conducted the one-to-one interviews in private meeting rooms in the hospitals in 2015. Each interview lasted approximately 30 min and was audio-recorded, transcribed, and anonymised. The interviews were based on an interview guide, which included themes concerning the nurses’ experiences of being interrupted, such as their definition of an interruption, how they experienced interruptions, the significance of interruptions for patient safety, and personal, ward-based, and hospital-based strategies to prevent and avoid interruptions.

### Data analysis

We analysed the semi-structured interviews using qualitative content analyses described by Graneheim and Lundman [26]. The analysis was based on inductive reasoning [27, 28], where an open-minded approach was adopted to identify meaningful subjects and extend an interpretive level of understanding towards nurses’ experiences. Each member of the research team read and listened to the transcripts independently to obtain a sense of the whole. We reflected on the content and analysed how the nurses understood and handled interruptions during medicine rounds in hospitals. We then broke the text into smaller meaning units, which were condensed and used to describe the narratives. Each condensed meaning unit was abstracted and interpreted to obtain the underlying meaning. We further abstracted these condensed meaning units and labelled them with themes, which were understood in relation to the context [26]. We re-read the interviews and checked whether all contents in relation to nurses’ experiences of interruption when administering medicines had been covered.

Considering the context, the meaning units were condensed into a description close to the text and the manifested content; thereafter, it was revised to reflect the interpretation of the underlying meaning, that is, the latent content, which was distant from the text but close to the nurses’ experiences [26, 28]. The analysis was not linear but a back-and-forth process between the whole and the parts of the text [26]. Throughout the analysis, the research team met several times to discuss the results of the analysis and reach a consensus. The goal was to reach a reasonable explanation of the underlying meaning of the text [26, 28]. The challenge in the movement from one stage to another was to stick to the essence of the nurses’ responses. During the analysis, a recurring theme — the latent content — was developed and the main theme ‘Working in a minefield’ was formulated. We considered data saturation to have been achieved when no new information arose from re-reading the transcriptions [25, 29, 30]. The themes are presented in the Results section along with quotations, which were grounded in the nurses’ experiences and representative of the participants’ responses.

## Results

This qualitative interview study elaborates on the results from Alteren et al.’s observational study [1]. Thirty-two nurses responsible for medicine rounds agreed to participate in the observational study. The nurses’ (30 female and 2 male) ages ranged from 22 to 68 years (mean 39 years). In the qualitative interview study, 19 of the 32 nurses were interviewed: seven from each surgical ward and five from the medical ward. No new information emerged after the first 16 interviews [25]. The nurses’ (17 female and two male) ages ranged from 22 to 60 years, with a mean of 39 years.

Six hundred and forty-four condensed meaning units were identified and used to generate themes. These themes were further condensed and abstracted to a general description of the research’s main theme (Table 2).

**Table 2:** Content analysis: From meaning units to main theme: ‘Working in a minefield’

Meaning unit	Condensed meaning unit Description close to the text	Condensed meaning unit Interpretation of the underlying meaning	Theme
If a patient needs help, I either have to find a nurse to look after the medicine trolley, take the medicine trolley back to the medicine room, or bring it into the patient’s room.	I have to reorganize my work and make sure the medicine trolley is safe	Dealing with unscheduled work	“Working in environments of interruptions”
When I am interrupted in the medicine room, I must check the doses again	If I’m being interrupted when preparing the medicine round, I have to re-check the doses	Unpredictability in the medicine round	“Working in environments of interruptions”
Giving water is a part of administering medicines. I always bring water with me when I administer medicine.	Bringing water with me is a part of administering medicine	Individual definitions of medicine administration	“Personal coping strategies”
One of my strategies is to make a mental note or a checklist. I have a small book in my pocket, where I have written when the patient shall have the medicine, and then I cross off	I have a mind-set and I write in a book when the patient shall have the medicine, and after giving the medicine, I cross it off	Practical strategies	“Personal coping strategies”
I do not get the time needed to help patients take all the tablets. I often trust that the patients take them by themselves. Often the patient needs help, so after the ward round, I can see that the tablets still are on the nightstand	When I am responsible for the ward round, there is little time to help the patient. I trust the patient and leave the tablets on the nightstand.	Adapting administration of medicines to the work-situation	“Personal coping strategies”
The hospital has not devised any strategies. It has not facilitated any protection of nurses from interruptions when administering medicines	The management has not devised any strategies to prevent the nurses from being interrupted	Lack of leadership	“Management-related strategies”
It seems that everyone knows that interruptions are a problem, but no one has any solutions. We tell the management that we have been in a hurry, and we see that there have been mistakes, for example with mixtures. We see that drip hangs up too long or that the patient should have had it at another time	Everyone knows that interruptions are a problem. No one has any solutions. We tell the management that we are in a hurry and that mistakes are happening	Taking interruptions seriously	“Management-related strategies”
In the ward, there could be more focus on speaking in departmental meetings about how to avoid interruptions and make an agreement on how to avoid being interrupted. For example, that we are not interrupted by others who convey a blood pressure or weight, but write the numbers on a note	In departmental meetings there could be increased focus on how to avoid interruptions and reach agreements on how to interact with each other to avoid interruptions	Making routines for administering medicines	“Management-related strategies”

The main theme, ‘Working in a minefield’, summarised the essence of how the nurses understood and handled interruptions during medicine rounds in hospitals. The nurses worked under high risk and dealt with unscheduled work and unpredictability in the medicines round. They overcame obstacles by developing strategies and adapting the work within the ward’s systems, characterized by lack of any measures to reduce interruptions and distractions. The handling of interruptions was left to the professional judgement of the individual nurse. Their strategies and adaptations protected them against risk and helped them maintain concentration to ensure the quality and safety of medicine administration. The three themes are presented under following headings: ‘Working in environments of interruptions’, ‘Personal coping strategies’, and ‘Management-related strategies’.

### Working in environments of interruptions

Working in environments of interruptions meant that the nurses had to deal with unscheduled work and demands. The daily routines of patients, colleagues, and the wards created this unpredictability. As nurses attended to the patients assigned to them, several unplanned and unanticipated events would occur, and they had to re-organise their work plans. For example, while a nurse attended to their patient, another patient would request help with the toilet, getting their pillow cushioned, or ask for a slice of bread or something to drink. When confronted with such situations, nurses would prioritise either the continuation of medicine administration or interrupt themselves, leave the trolley, and perform the other nursing duties. They made such decisions based on the patients’ needs and the ward’s daily routines and activities:

*There is something all the time. During the medicine round, I must prevent pressure ulcers with lubrication and relief. It is important, but not so important when I administer medicine. I need to focus on one thing at a time.* (Nurse 9).

Another example of an unpredictable event was patients asking for medicine from a nurse was not responsible for that patient. The two teams were organised such that patients in one room belonged to different teams, but the patients were unaware of this arrangement. When a nurse entered the room to distribute medicine to their patient, the patient in the neighbouring bed would also request for their medicine. This would distract the nurse, and they, in addition to giving medicine to the patient they are responsible for, would explain to the patient in the neighbouring bed which other nurse was responsible for their medicine. Sometimes the query could be about analgesics or intravenous treatment. If the nurse helped the patient in the neighbouring bed or tried to find the responsible nurse, it delayed the administration of medicine to their patient. Consequently, patients would not receive medicine at the right time. Moreover, the nurses on the other team would be disturbed and would have to interrupt their medicine rounds to come and help the patient. Another unpredictability was when nurses’ colleagues, such as other nurses, nurse assistants, medical doctors, and physiotherapists interrupted them by tapping the door of the medicine room or asking questions unrelated to medicine administration.

Sometimes during medicine administration, nurses realised that their colleagues were overwhelmed with too much work. In such situations, they had to decide whether to leave the medicine round and help their colleagues by, for example, answering the phone or taking a patient alarm, or prioritise the administration of medicines to their patients, and not assist their colleagues or other patients:

*I give priority to administer medicines and cannot help the patient with toilet. In these situations, I ask the patient to pull the alarm bell, so someone else can help him or her.* (Nurse 10).

The nurses said that they could lose concentration if they had to leave the medicine round to perform unscheduled work, such as answering the phone or taking a patient alarm call.

In other instances, the nurses experienced unscheduled work when they had to return to the medicine room because the medicine was placed incorrectly in the dose distribution system, or some medicine was missing from the trolley. Unforeseen changes in the wards’ daily routines also resulted in the loss of concentration when administering medicines. Examples were time pressures, unforeseen changes, or when the doctor arrived for the ward round earlier than predicted:

*Suddenly, the doctors can come before they are supposed to. Then they expect me to have completed the medicine round. I am not necessarily ready.* (Nurse 1).

*I can be told that bed three in room two shall have ketobemidone hydrochloride (synthetic opioid analgesic, not available in many countries) without knowing the patient’s name. In surgical wards, the patients are moved all the time, and when I enter the room, it may not be the right patient lying in bed three. The patient might have been moved in the meantime*. (Nurse 14).

### Personal coping strategies

The nurses administered medicines as per the ward’s established routines and activities. To do so, nurses individualised coping strategies, such as creating individual definitions of the concept of medicine administration, developing practical strategies, and adapting to the work-situation.

In the nurses’ personal definitions of medicine administration, they included nursing duties which were not part of the medicine administration procedure. While these varied from nurse to nurse, communicating with the patients, following patients to the toilet, bringing patients water, or answering questions concerning blood samples were examples of nursing duties encompassed within many of their personal definitions of medicine administration procedures. When the nurses worked within their definitions of medicine administration, they did not perceive the additional tasks as interruptions. However, the nurses considered certain tasks as interruptions if the tasks were too many in number or they fell outside their accepted definitions. This was the case even if the additional tasks were originally accepted as being within their definitions of medicine administration procedures.

Normally, the administration of medicines should be completed within a defined period set by the ward. To stay within the period and handle the time-pressure, the nurses developed different practical strategies to ensure the quality and safety of medicine administration:

*I put the medicines and drips in order. I have a solid pattern I follow. When I get disturbed, I get out of the count and I have to start to order again*. (Nurse 17).

To avoid leaving the trolley during the medicine round, nurses would bring a jug of water and glasses on the trolley. The nurses found that patients requested for analgesics at night. To prevent interruptions and meet the patients’ needs, the nurse brought analgesics with them as well. Another example was during the day shift when the medicine journal was required to be completed. According to the ward’s established routine and the nurses’ goal, medicines had to be given before the ward round when the doctors needed to see the medicine journal. When nurses were interrupted, medicine administration was postponed and not completed before the doctor’s ward rounds. As a strategy to complete the medicine administration, the nurses copied the medicine journals. However, even if they adopted personal strategies and adapted their administration to the organisational system by, for example, ensuring that the patient took the medicine and avoiding other unrelated tasks, they were unable to complete the administration of medicines within the prescribed period:

*I do not get the time needed to help patients taking all tablets. I often trust that patients take the tablets themselves. Often the patient needs help, so after the visit, I can see that the tablets still are on the nightstand*. (Nurse 4).

The nurses reported that they could not always give the patients their infusions at the right time. As a result, they adapted the administration of medicines to the work-situation:

*If the patient needs antibiotics, right time is important. I always give the infusion with antibiotics before other infusions.* (Nurse 3).

*Law requires double signing. In summertime, and when we are too few people at work, we are not able to double sign for controlled drugs. Then I have to leave the medicine room to find colleagues, who are busy. We have warned the management that it is difficult to get double signatures. The managements are now accepting lack of double signature.* (Nurse 10).

### Management-related strategies

The nurses experienced lack of leadership and reported that their managers were not taking interruptions and their consequences on patient safety seriously enough. The nurses indicated that guidelines for administering medicines increased concentration and reduced interruptions, which ultimately safeguarded patients.

The nurses also reported that they informed the management when they were in a hurry or when they observed mistakes being made, such as tablets being left on the nightstand and intravenous medicines not being given at the right time. They felt that neither the hospital nor the ward tried to protect them from interruptions. The management was aware of the interruptions and their effects on the administration of medicine, but they took no action, which gave the nurses the impression that the management was not giving due consideration to interruptions. A nurse said:

*To be able to implement good and preventative measures, the management must take interruptions seriously*. (Nurse 1)

Therefore, it was left to the individual nurses to protect themselves against interruptions. The nurses communicated to their managers in formal and informal meetings about the interruptions they experienced when administering medicines and the dangers of such interruptions to patients’ safety, and they also provided suggestions on how the interruptions could be avoided. The nurses suggested that guidelines and agreements be developed on how to avoid interruptions, an example being the double-checking of dose calculations and controlled drugs, which could enhance patient safety. The nurses reported that instead of taking responsibility and finding solutions, the management merely admitted to the absence of the double-checking procedure.

The nurses also demanded management’s engagement in writing good and practical guidelines through discussions or in revising failing guidelines. Examples include guidelines for procedures when patients are given the wrong medicine, not given the medicine they are supposed to get, or not given medicine at the right time. The nurses said that it was the management’s responsibility to take the initiative and change the routines.

## Discussion

The main theme of this study, ‘working in a minefield’ describe nurses’ experiences of working in environments where they were frequently interrupted and the strategies they adopted to effectively reduce interruptions and improve safety during administration of medicines in hospitals.

Nurses work in environments with interruptions where they have to deal with unscheduled work and patient demands [1, 19]. Additionally, our study shows that nurses are expected to adhere to the organisation’s guidelines on how to handle interruptions. Irrespective of the time of day or day of the week, nurses face complex situations and a mixture of varied and often competing demands involving numerous scheduled and unscheduled tasks, which should be completed within the shift [19]. This causes situations that lead nurses to make errors, which consequently render medicine administration unsafe [10, 11].

The findings of our study showed that when the nurses faced interruptions, they failed to give medicine to the patients at the right time and would leave the medicine on the nightstand — this is unsafe and threatens patient safety. As per Norwegian regulations, “medicine is not given before it’s taken” [31] as other patients may take the medicine, the medicine may not be taken at the right time or at all, or it may be consumed with incompatible medicines or food (e.g., tetracyclines could be taken with milk). Our study highlights that unforeseen work and demands delayed medicine administration and affected the preparation of intravenous medicines. This occurred even when the nurses prioritised their tasks considering the consequences it might have for patients.

The nurses’ primary goal was to ensure patient safety during medicine administration. Through experience, they developed personal strategies to ensure this. Sitterding [21] described the development of individual interruption-handling strategies in which multi-tasking or engagement was the nurses’ preferred strategy. In our study, nurses did not describe the personal strategies as guidelines in the ward but as individual measures to reduce errors. Although the hospital policy and ward’s routines helped them stay focused on the procedure, they also contributed to interruptions. The nurses therefore took measures to reduce the consequences of the interruptions. They were afraid of making mistakes, and therefore ensured, among other things, that they double-checked the medicine against the medicine journal. Did they devise individual strategies to be able to deal with unpredictable work-situations? The documentation of small differences in behaviours and actions after interruptions may explain individualised adaptation among nurses with extensive experiences [1].

Adaptation over time can hide an underlying problem, which assumes a life of its own, with unknown and unfortunate consequences, thereby threatening patient safety. The nurses in our study stated that interruptions were challenging in the ward environment and that measures could be implemented to reduce these. They reported to the ward management about mistakes, which they saw or made and gave suggestions for measures to be taken; however, they found that management did not follow-up. Nurses reported the management’s lack of concern over improving routines for administering medicines. The nurses interpreted this as management’s acceptance of interruptions and their belief that individual nurses must protect themselves against interruptions and consequent errors.

Lack of action from the management contributed to the continuation of practices, which the practitioners had no control over. A practice that threatens patient safety is a challenge, which the ward management must address. The ward management is responsible for the development of best practices, both up and down in the hospital hierarchy. Previous research has found that fewer interruptions were found to be related to medicine tasks, such as questions about another patient, requests from patients, and questions/discussions about treatment or equipment [4]. The nurses’ colleagues, other staff, and the nurses themselves were the most frequent sources of interruptions during medicine administration [1, 17, 18].

The findings of previous studies [1, 4, 17, 18] indicate that incidences of interruptions can be reduced. Systematic reviews assessed the effectiveness of strategies aimed at reducing interruptions during medicine administration [12–14] and found weak evidence of the effectiveness of strategies such as quiet zones, checklists, and vests and very limited or no evidence of their effectiveness in reducing medicine errors [13]. The results of our study suggest that management should focus on developing measures that go beyond practical solutions such as quiet zones and vests, and create conditions contributing to the development of a culture wherein interruptions are tackled in order to collectively develop and improve nurses’ working conditions. Managers should ask themselves: What kind of practice do we want in our ward? Discussions on best practices can shift the focus from a personal to a collective responsibility, which can help create a better organisation that cares for its staff by reducing interruptions by colleagues, patients, and relatives. Such management contribution can ensure patient safety and create safe working conditions for nurses administering medicines [32, 33].

### Strengths and limitations

In this study, we elaborated on the results from Alteren et al.’s [1] study by deepening the understanding of what lies beneath the surface of nurses’ working environments. These two studies complement each other, strengthen the findings, and create a better understanding of the interruptions faced during medicine administration.

A strength of this study is that it offers information from the nurses’ point of view regarding their working environments wherein they are frequently interrupted. Furthermore, their strategies to reduce interruptions are described. This knowledge is valuable in the discussion of best practices and patient safety, which demand cultural changes at all service levels [1].

Most participants were highly experienced nurses who were interested, engaged, and committed to the subject and willing to contribute during the interviews. They did not hesitate to share information about the challenging situations they encountered; the errors they made or observed, such as leaving medicines on the nightstand and lack of double-checks; and the consequences which the interruptions and ward organisation could have on patient safety, such as medicines being given at the wrong time. However, we were unable to obtain the managers’ perspectives and their responses to the criticisms levelled against them by the nurses.

During the interviews, the researchers summarised the main themes of the conversation to ensure that the participants understood the content and scope of the research [34]. Another strength of this study is that the authors discussed and agreed upon the condensed meaning units and themes at various levels, and concurred on the condensation and abstraction of the themes to a general description of the research’s main theme, which ensured transparency and contributed to the credibility and validity of the study’s findings [27]. The context in which the research was conducted, how the participants were selected, characteristics of the participants, and how data were collected and analysed were described. Moreover, the results were presented in the participants’ own words. The discussions within the author group about the method, findings, and analysis also helped strengthen the validity of the study. However, the nurses were highly experienced, so the study findings may have limited transferability to less experienced nurses outside Norway.

## Conclusions

This study offers insights into nurses’ coping strategies when administering medicines in hospital environments characterised by constant interruptions. To handle these interruptions, nurses made their own adjustments to reduce errors and adapted their work around the interruptions. Ensuring safe medicine administration is a continuing task. The management must prioritise reducing interruptions and take the lead in developing best practices, such as ensuring safe ward conditions for nurses to administer medicines, which in turn will increase patient safety. Further research on managements’ responsibility and facilitation is needed to develop best practices and increase patient safety.

### Clinical implications

The knowledge and experiences of health personnel can provide input for professional development and education in surgical and medical wards. The findings of our study contribute to discussions about medicine administration and its consequences for patient safety. Managers are responsible for facilitating safe working conditions, best practices, and patient safety. Without organisational change, medicines will continue to be administered late or be left on nightstands. The nurses cannot avoid making mistakes; therefore, dialogue between the management and nurses will improve the safety of working conditions, reduce unnecessary interruptions, and facilitate best practices.

## Data Availability

The datasets generated and/or analysed during the current study are not publicly available due to permission has not been applied for from neither the participants nor due to Norwegian privacy legislation and the form signed by the participants about the study’s privacy. The data generated are available from the corresponding author on reasonable request.
